# P02.20. Mindfulness based tinnitus stress reduction (MBTSR) pilot study: a symptom perception shift program

**DOI:** 10.1186/1472-6882-12-S1-P76

**Published:** 2012-06-12

**Authors:** J Gans

**Affiliations:** 1 University of California, San Francisco, San Francisco, USA

## Purpose

Estimates suggest that as many as 50 million people in the United States experience tinnitus to some degree. Yet for 16 million of these Americans, tinnitus is a chronic condition significantly impacting quality of life. Sleep disorder, depression, and anxiety are common co-occurring symptoms. The exact physiological causes of tinnitus are unknown making it difficult to treat with only variable success. This pilot study aims to investigate whether a novel mind-body intervention, Mindfulness Based Tinnitus Stress Reduction (MBTSR), may be a beneficial treatment for chronic tinnitus.

## Methods

Eight tinnitus patients who had previously received Tinnitus Counseling (standard of care) at the UCSF Audiology Clinic participated. Tinnitus symptom activity and discomfort as well as psychological outcomes were assessed by self-report questionnaires. Both quantitative and qualitative data were gathered. The primary outcome measure was the Tinnitus Handicap Inventory (THI) measuring subjective or perceived handicap or reaction. Change in mindfulness, Health Related Quality of Life (HRQoL), and other clinical symptoms such as anxiety and depression were also assessed. The secondary outcome measures included a Tinnitus Visual Analogue Scale (VAS), a measure of tinnitus awareness, SF36 Health Survey (SF-36), the Symptom Checklist-90-Revised (SCL-90-R), Hospital Anxiety and Depression Scale (HADS), and the Five Facet Mindfulness Questionnaire (FFMQ).

## Results

Results indicate that effect sizes, if supported by a larger study, may be clinically significant and demonstrated a decrease in subjective tinnitus handicap, awareness, and annoyance, reduction in depression and phobic anxiety, improvement in the mindfulness facet of non-judging, with increases in social functioning and overall mental health. Change scores across all study measures moved in hypothesized directions.

## Conclusion

This pilot study provides preliminary evidence that an 8-week MBTSR program may be a promising intervention for treating chronic tinnitus and its comorbid symptoms. These promising findings warrant further investigation using a randomized controlled trial.

